# Benchmarking the robustness of the correct identification of flexible 3D objects using common machine learning models

**DOI:** 10.1016/j.patter.2024.101147

**Published:** 2025-01-10

**Authors:** Yang Zhang, Andreas Vitalis

**Affiliations:** 1 Department of Biochemistry, University of Zurich, 8057 Zurich, Switzerland

**Keywords:** flexible object recognition, machine learning, benchmark set, molecular dynamics, featurization, structural representation, molecular science, 3D spatial data

## Abstract

True three-dimensional (3D) data are prevalent in domains such as molecular science or computer vision. In these data, machine learning models are often asked to identify objects subject to intrinsic flexibility. Our study introduces two datasets from molecular science to assess the classification robustness of common model/feature combinations. Molecules are flexible, and shapes alone offer intra-class heterogeneities that yield a high risk for confusions. By blocking training and test sets to reduce overlap, we establish a baseline requiring the trained models to abstract from shape. As training data coverage grows, all tested architectures perform better on unseen data with reduced overfitting. Empirically, 2D embeddings of voxelized data produced the best-performing models. Evidently, both featurization and task-appropriate model design are of continued importance, the latter point reinforced by comparisons to recent, more specialized models. Finally, we show that the shape abstraction learned from database samples extends to samples that are evolving explicitly in time.

## Highlights

• Recognizing different poses of flexible objects can be challenging
• We derive benchmark sets from molecular science as prototypical examples
• Representation continues to matter in modern machine learning
• Flexibility across proteins and flexibility in time are understood and compatible

## The bigger picture

Many natural objects have intrinsic flexibility, for example, through articulated joints in living beings such as humans. In applications like autonomous vehicles, it is important that a class of object captured through imaging devices in either 2D or 3D is safely identified. Differences caused by motion and flexibility are often confounded by intrinsic differences, as seen, for example, in different plants of the same type of tree. Thus, reliably recognizing such objects is a challenging problem. Our study creates a bridge to this problem scope from molecular science by offering datasets for benchmarking methods trying to solve this recognition task. Molecules are flexible and offer many unequivocal classes that can “look” very similar. We were interested in how well modern machine learning methods perform in this task when they have to rely on spatial information alone. Taking a dataset from molecular science cures some technical issues seen with imaging data, such as differences in scale, resolution, and ambiguous labels. Our research shows that the exact way in which the spatial information is encoded continues to be important, and this holds for both accuracy and transferability. The latter can be thought of as a proxy for the appropriateness and generalizability of the strategy a given model learns. Transferability is the biggest concern in fields where there are limited and often non-extensible amounts of data, such as drug discovery, digital humanities, or financial modeling, and we touch upon the implications of our results for applications of machine learning in such a setting.

## Teaser

Molecular science benchmark sets (FEater) are introduced for machine learning tasks involving flexible object recognition. The impact of differences in featurization and model architectures on both the expected accuracy and the achievable transferability is discussed. In addition, numerical evaluations of the performance of the workflows are provided, and the compatibility of the flexibility observed over time (molecular dynamics) with the heterogeneity found across independent observations (structural databases) is evaluated.

## Introduction

The spread of three-dimensional (3D) sensors in recent years^1^^,^^2^^,^^3^ has significantly advanced the field of 3D data acquisition. Combined with the development of dedicated 3D reconstruction algorithms,^4^^,^^5^ this technological progress has facilitated the creation of extensive 3D object datasets that vary in focus, encompassing rigid objects,^6^ soft objects,^7^^,^^8^ images with embedded depth maps (RGB-D),^9^ and 3D scenes.^10^ This expansion of resources has been instrumental for innovating 3D-based tasks such as object recognition, reconstruction, and semantic segmentation. Point clouds, as a prevalent form of 3D data representation, express the object or scene as an unordered set of points with distinct local and global features. Various techniques designed to work directly with point clouds have emerged, such as point-cloud completion, refinement, and object recognition.^11^^,^^12^^,^^13^ Surface meshes are commonly used to characterize the exterior surfaces of objects and are dominantly used in computer graphics for rendering and storing 3D models.^14^ By representing objects in a volumetric form or mapping visible surface geometries to a volumetric space, 3D voxels also encode the geometries of objects for recognition tasks.^6^^,^^15^ Recent developments in the detection of human motion, path planning for robots, or self-driving cars^3^^,^^16^^,^^17^ demonstrate the potential applicability of 3D-based training methods in problems that have to deal with dynamic (continuously changeable) content.

In molecular science, machine learning (ML) promises powerful tools for drug discovery tasks due to its applicability to problems that have ample data but lack a clear mathematical model.^18^^,^^19^^,^^20^ Parameter-rich ML models, commonly known as "deep learning" approaches, have gained fame for their ability to handle largely unprocessed input data and find relevant features on the fly. Even so, the handcrafted definition/engineering of features remains a critical step of many conventional drug discovery pipelines.^21^^,^^22^ Features must carry the necessary task-specific information, which limits the transferability of the models and entails a lack of universally applicable guidelines during feature design. Geometric deep learning (GDL) aligns neural networks with both Euclidean and non-Euclidean domains, such as graphs, manifolds, and meshes,^23^ to facilitate the recognition of symmetries and needed invariances. Recent progress in computing hardware has allowed several GDL algorithms to demonstrate significant improvement on tasks such as protein-ligand affinity prediction,^24^^,^^25^^,^^26^ protein binding site identification,^27^ protein interaction prediction,^28^^,^^29^^,^^30^ and protein structure prediction.^31^^,^^32^^,^^33^^,^^34^

**Figure 1:**
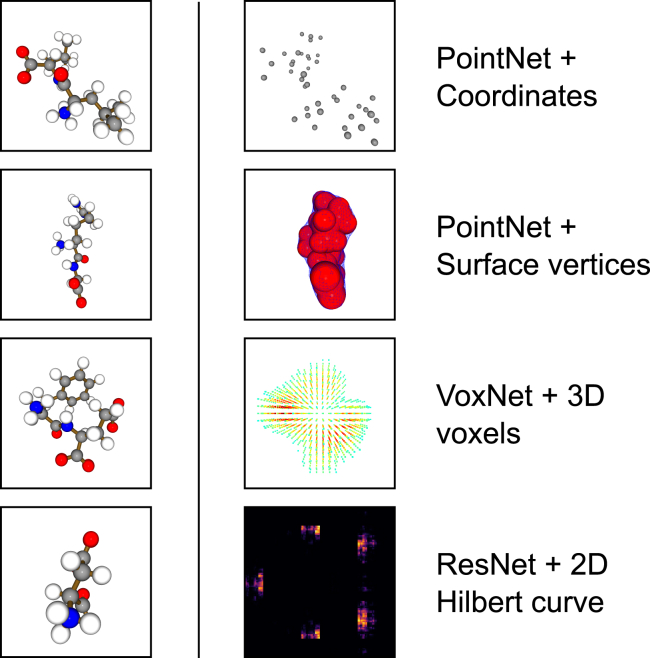
Overview of the primary combinations of features and models tested

A 3D molecule is generally represented by a (3D) graph formed by vertices (atoms) and edges (bonds). Given the scaffold of a molecule, the aforementioned 3D representations, such as coordinates (point clouds), surfaces (polygon meshes), and density (3D voxels) (Figure 1), are routinely derived for computational chemistry research. When bond and atom order are not of interest, atomic coordinates can be viewed as point clouds. The solvent-excluded surface reflects the envelope of a molecule in 3D space. It is normally obtained as a triangle mesh from an accessibility grid with the help of atomic radii,^35^ and point-cloud-based methods are applicable to the vertices of this mesh.

Canonical point-cloud features are encoded by local^36^^,^^37^ and/or global characteristics.^38^ The growth in available 3D data and the availability of consumer-level 3D cameras have increased the emphasis on the direct processing of 3D point clouds. Reducing the specificity requirements of input features is a central aim. For example, PointNet and PointNet++^11^^,^^12^ provide a network structure that ensures the permutation invariance of the points. PMP-Net++^39^ transforms/augments incomplete point clouds by optimal point movement paths (PMPs) to restore the integrity of the represented scenes. DensePCR^40^ is able to hierarchically increase the resolution of crude point clouds.

Voxel-based representations are native to molecular science as well: the electron density map is an experimentally observable, voxel-based representation characterizing the probability distribution of electrons in the molecule. More broadly, 3D voxels offer a general route to the explicit encoding of 3D geometries, and 3D convolutional neural networks (3D-CNNs) can extract and learn the relevant features during training. 3DShapeNets^6^ converts the 2.5D depth map to a 3D volumetric grid for object recognition and reconstruction. Similarly, VoxNet^41^ transforms point clouds into occupancy grids for real-time object recognition. The rapid escalation in computational demands driven by increasing resolution^15^ and the fact that macroscopic objects are not perceivable as volumetric objects due to occlusion, e.g., filled vs. hollow containers, are hindrances for voxel-based approaches. In molecular science, perspective projections and related issues are not major issues. By extracting the 3D structure of the binding site, featurized 3D voxels are now widely used in ligand binding affinity prediction.^24^^,^^25^^,^^26^^,^^42^^,^^43^^,^^44^ They are also applied for protein-protein interaction prediction,^30^ binding site identification,^27^^,^^45^ and molecular docking.^46^ That said, most often, a combination of chemical properties and spatial relationships guides the predictions, and the majority of earlier ML-assisted drug discovery research thus relied on hand-extracted features passed to off-the-shelf classifiers with static structures.^47^^,^^48^

Many ML tools in drug design explicitly disregard the conformational flexibility of the entities involved. Knowledge of this flexibility is important yet highly contextual and challenging to capture.^49^ Just like macroscopic objects with articulated limbs, ligand binding sites are not rigid, nor can they be reshaped arbitrarily. Dynamic features have been proposed, e.g., in MDFP+,^50^ which combines 2D fingerprints with statistical distributions of properties from molecular dynamics (MD) trajectories such as solvent-accessible surface areas to predict free energy differences. Similar ideas have been pursued in recent years with different goals.^51^^,^^52^^,^^53^ Since physical atoms are in constant motion subject to topological constraints, molecules can be considered semi-rigid to soft objects. Comprehending their dynamicism with ML promises deeper insights into molecular processes that are accompanied by conformational (shape) changes.

However, in order to utilize this information meaningfully, it is paramount that object identity and flexibility can be perceived distinctly. Thus, in this work, we ask how efficiently a given model abstracts the shape changes of typical molecular building blocks while correctly recognizing their identities. We focused on the fundamental building blocks of proteins as an archetype and designed a pipeline to qualitatively measure the efficiency of models in abstracting from the different shapes these building blocks can attain. We constructed a dataset of 3D, residue-based fragments from experimental structures. Specifically, the FEater (flexibility and elasticity assessment tools for essential residues)-Single dataset contains 20 labels marking the 20 standard amino acids, while the FEater-Dual dataset contains 400 labels mapping to the 400 combinations of two-residue stretches. The latter not only contains many more classes but also dramatically increases the in-class heterogeneity and is expected to be much more challenging. The performance evaluation pipeline is straightforwardly extensible to other benchmark sets, and we demonstrate as much for ModelNet40.^6^ Here, we converted the protein-based fragments into four 3D-based representations. We show that by blocking the data through clustering, a stringent training baseline is established. We then trained general-purpose classifiers (Figure 1), systematically increasing the training data size and scope, to demonstrate that increasing data richness does result in systematic performance improvements, which is a hallmark of successful learning. We compare the methods’ effectiveness in classifying the labels, both among the basic models and to a number of more recent literature examples. The results highlight that both featurization and model design continue to matter. In the final part, we investigate how well models trained on the FEater datasets extrapolate to samples taken from MD simulations, i.e., explicitly time-dependent data.

## Methods

### FEater: A 3D dataset of molecular fragments

The 3D molecular training of ML models is typically a specialized, purpose-driven workflow. Such an approach can lead to implicit feature design and potential biases toward 3D shapes, making it difficult to assess a model’s generalizability. To address this challenge, we propose a standard workflow to establish a baseline performance that is independent of the specific application.

**Figure 2:**
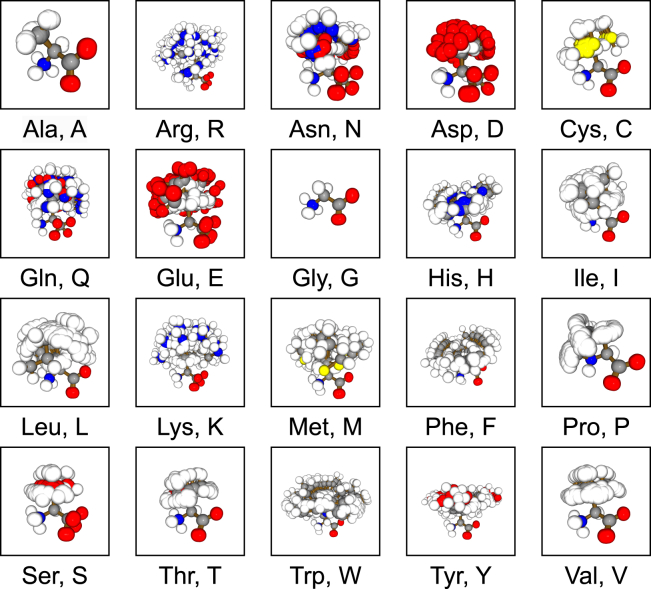
Overview of 100 aligned and superposed conformations of the 20 standard amino acids from the validation set Images are labeled with both 3-letter and 1-letter codes, which we use throughout the manuscript. The color indicates chemical elements (red: O, blue: N, gray: C, white: H, yellow: S). Individual examples for the two-residue case are shown in Figure S1.

**Figure 3:**
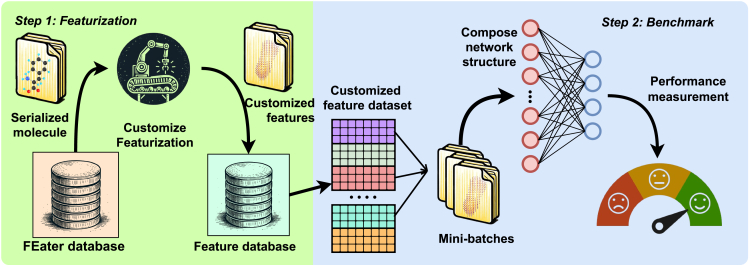
Illustration of the proposed workflow to measure model performance on customizable input data

Inspired by the MNIST dataset, which utilizes a simple, definitive task to benchmark learning algorithms, we present the FEater dataset to test how well a 3D shape encapsulates chemical identity for flexible and mutually similar moieties. There are two sub-datasets for different degrees of complexity: the FEater-Single (Figure 2) dataset encompasses single residues extracted from each protein structure and the FEater-Dual dataset extracts all the consecutive dipeptide stretches instead. By featurizing the molecular representation to a candidate model (Figure 3), the performance and convergence speed can be tested to compare the fitness of this combination quantitatively. Here, we converted the fragments into four different, 3D-based representations and trained general-purpose networks to serve as community benchmarks (Figure 1).

Since proteins are soft objects whose properties, in isolation, are rotation invariant, we expect this database to find use in the field of computer vision. Molecular fragments resemble objects with movable joints (animal or human poses) or tree-like topologies (plants). Importantly, the complexity in the FEater datasets is complementary to standard computer vision benchmark sets such as ModelNet.^6^ In the latter, the difficulty stems from heterogeneity that is not linked to motion, e.g., a sedan vs*.* a sports car, but the inter-class differences are almost all quite pronounced. Conversely, in FEater, all in-class differences are mappable to the same graph of covalent bonds and rotations around dihedral angles, but the inter-class differences, viewed spatially, are very small. As a result, models receiving explicit bond graphs as input serve as a positive control for classification but provide no insight into the ability of ML models to learn 3D shape abstraction. Because simple residues, Ala and Gly in particular, have limited numbers of degrees of freedom (Figure 2), we anticipate some redundancy in FEater-Single at the level of 3D conformations. Notably, the datasets are designed for model evaluation rather than specific biological tasks.

### FEater dataset construction

Folded proteins contain residues in many different conformational states. Here, we use the PDBBind (v.2020)^54^ general set, which contains 19,443 protein-ligand complexes, as the pool of residue fragments. The clear majority of residues are not in direct contact with ligands or cofactors, so the restriction of considering only proteins known to bind ligands is expected to be inconsequential while allowing us to benefit from PDBBind’s filtering, e.g., against low-resolution structures. In the end, 19,433 complexes were kept. Since the residues taken directly from PDBBind are incomplete (hydrogens are missing, terminal atoms are heterogeneous), we homogenized the data by adding missing atoms with CAMPARI (http://campari.sourceforge.net/v5) to restore the chemical integrity of blocks. Since this database focuses on fragment recognition, namely one or two consecutive residues, cysteines that are part of disulfide bonds were treated as dissociated, and the S-H atom was added. Histidines were all processed to be in the neutral form with the $N_{\delta }$ atom protonated. In raw form, FEater-Single encompasses 8,802,999 entries, and FEater-Dual contains 8,749,157 stretches. Importantly, the pool of two-residue fragments comfortably covers all 400 combinations of the 20 standard amino acids. We did not pre-align the data to remove rotational ambiguity and kept the original coordinates. The processes of fragment extraction are described in Algorithms S1 and S2. To balance the classes in the dataset, upper-bound cutoffs are set to 5,000 entries per class for the training set and 1,000 for the validation and test sets. Consequently, the serialized, balanced FEater-Single dataset includes 100,000 entries for the training set and 20,000 for the validation and test sets. FEater-Dual contains 1,929,581 entries in the training set, 393,405 in the validation set, and 393,421 entries in the test sets. These sets are subsampled further in the tests below. The topologies of the fragments are stored in the same hierarchical data format (HDF) file and named by either 3- or 6-letter (2 × 3-letter) codes.

### Surface mesh generation

SiESTA-Surf^35^^,^^55^ was used to generate the surface mesh of the residues on the GPU. The grid spacing was set to 0.35 Å for the balance between resolution and dataset size, and the step number of Laplacian smoothing was set to 1 to avoid losing finer local features. The number of slices was 300 to allocate enough memory.

### Voxel generation

To prevent a hidden encoding of atom types, we used a uniform property value equal to 1, regardless of atom type, to estimate volumetric density. The bounding box size is 16 Å to accommodate residues of different sizes and orientations. We chose 32×32×32 voxels to balance the cost, size, and resolution (0.5 Å). For the voxelization of atoms, we used distance-based Gaussian mapping to generate 3D voxels, similar to DeepRank,^30^ and CUDA was used to accelerate the computation (detailed in supplemental methods, voxel generation, and Algorithm S3). The cutoff and smoothness factor (σ) were set to 12 and 1 Å, respectively. Before the computation, the center of the geometries of fragments was moved to the center of the bounding box.

### 2D Hilbert image generation

Space-filling curves^56^ are continuous lines to systematically span a space of a certain dimensionality. Hilbert curves,^57^^,^^58^ a specific type of space-filling curve, maintain the spatial proximity of points during a change in dimension. This characteristic is particularly useful for reducing 3D voxel data into 1D vectors, which can then be mapped back into 2D images, preserving much of the original spatial information. The voxel lattices of size 32×32×32 are represented by fifth-order 3D Hilbert curves ($2^{3\cdot 5}$), which do not directly correspond to 2D Hilbert curves due to the point count, falling between the seventh order ($2^{2\cdot 7}$) and eighth order ($2^{2\cdot 8}$) of the curve. In the end, we first performed downsampling on the 1D vector, mapped to 2D Hilbert curves of seventh order (128×128), and finally treated them as 2D images (see Algorithm S4).

### Construction of the baseline dataset

We decided to use a data-driven procedure to block the test and training sets, i.e., to try to reduce the conformational overlap between them. To do so, every class of the two datasets was processed individually following a two-step clustering. Up to 5,000 samples were randomly selected from the raw dataset and superposed using all heavy atoms. The pairwise root-mean-square deviation (RMSD) was calculated, and an initial clustering was performed using the Butina algorithm^59^ with a threshold of 75% of the mean RMSD. The clusters were sorted by size, and samples from the bottom 25% of these were added exclusively to the test set. For the remainder, in a second step, the data from larger clusters were split as follows. The samples of a given cluster (if there were more than 30) were embedded into two dimensions using t-distributed stochastic neighbor embedding (t-SNE)^60^ with the pairwise RMSD as the high-dimensional metric, as before. This was followed by agglomerative clustering into 10 subclusters. Finally, size permitting, 10 or fewer samples were randomly selected from each subcluster and ordered based on the size of their parent subcluster. To ensure the balance of the training and test datasets in each cluster, up to 10 conformations (from the first 2/3 conformers) were put into the training dataset, and up to 5 conformations (from the latter 1/3 conformers) were put into the test set. In both stages of this procedure, we populated the test set specifically with lower-likelihood conformations. That said, this heuristic approach is limited 2-fold: first, there can be too little diversity (compare Ala or Gly in Figure 2) for meaningful splits to occur, and second, some clusters might be in the top 75% by size but have fewer than 30 samples. In these cases, size permitting, a random 2:1 split of, at most, 15 samples was added to the training and test sets, respectively, which is a limitation.

### Feature data storage

**Figure 4:**
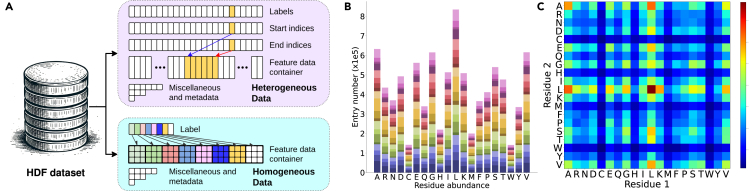
Structure of FEater (A) Storage of both heterogeneous and homogeneous feature representations. (B) Residue population distribution in the FEater-Dual dataset. The abundance of the second residue is encoded by different colors within each bar. The distribution in the FEater-Single dataset resembles the absolute height of bars. (C) Abundance of pairs in the FEater-Dual dataset (range from 1,544 to 78,499). There is no specific pairwise depletion. Instead, the lowest abundances are due to the rarest residue types (M, H, C, W).

The protein complexes give rise to over 17 million fragments, and file-based storage poses significant challenges to the stability of the host system. Hence, the HDF is utilized for the storage of various types of feature data. For homogeneous data with a fixed size, such as voxels (32×32×32) and images (128×128), each entry is stored as a slice of the HDF5 dataset. For partially heterogeneous data, such as coordinates and surface meshes, entries are concatenated along the first dimension because the second dimension is fixed. To allow the navigation of entries, the start and end indices are recorded as auxiliary datasets (Figure 4A).

Four built-in data loaders are implemented to facilitate the use of the dataset with multi-process pool acceleration. The benchmarks for data retrieval utilize two subsets with a sample size of 50,000 samples. The batch size was set to 128, and each run was repeated 10 times.

### Model selection

PointNet^11^ was chosen as the baseline model for point-cloud representations, including raw atomic coordinates and surface vertices. VoxNet^41^ and ResNet-18^61^ were deployed for 3D voxels and 2D Hilbert curves, respectively. Both 3D voxel and 2D image representations have only a single input feature channel. Notably, in VoxNet, following initial tests, we replaced the standard rectified linear units with the parametric version (PReLU) with one trainable parameter. The output class numbers are adjusted to either 20 or 400, depending on the training dataset.

For further benchmarking of the community models, the PointNet++ single-scale grouping (SSG) model,^12^ dynamic graph CNN (DGCNN),^62^ and PAConv^63^ were selected for point clouds. Two additional 3D CNN-based models, both originating from molecular science, were deployed to process 3D voxels: a model architecture from DeepRank,^64^ which we term “DeepRank-CNN” in the following, and Gnina-2018.^42^ We note that DeepRank is a broader framework, but here we only evaluated a fixed-size instantiation of its voxel-based CNN, which received the same voxel-based features as other CNNs as input.

Lastly, we picked ConvNeXt,^65^ isotropic ConvNeXt, Swin transformer,^66^ and vision transformer^67^ (ViT) as representative of modern CNN- and transformer-based image models. Notably, the number of sampled points in the first abstraction layer of PointNet++ is set to 24 for the training on the raw coordinates (one residue) because the default value of 32 exceeds the target number of points (24) in this scenario. Given that the absolute densities differ between atomic coordinates and surface points, we used multi-scale grouping with two distinct radii each for hierarchical perception in PointNet++: 1.75 and 3.6 for coordinates and 0.5 and 1.0 for surfaces.

### Model training

For point clouds, the points are first shifted by subtracting the minimum value of each coordinate axis before being either padded (if too few) or subsampled to a target number (if too many). To avoid latent association of features with the order of points, they are shuffled before being fed to the classifiers. For raw coordinates, the target numbers are 24 and 42 points for FEater-Single and FEater-Dual, respectively, while for surface vertices, the target number is 1500. All models were trained with the following scheme if not specified otherwise. The cross-entropy loss function and the Adam optimizer with momentum factors of 0.9 and 0.999 were used. The initial learning rate was set to 0.001 and rescaled every 30 epochs by a factor of 0.5. All models were trained for 120 epochs with a mini-batch size of 64. The initialization of the parameters for convolutional layers followed the Kaiming normal distribution.^68^ If the accuracy on the test dataset exceeded 99.5%, then the training was terminated. In some cases, to avoid overshooting, the initial learning rate was set to $10^{-4}$ instead: for PointNet++ (two residue, surfaces only), Gnina (two residue), ConvNeXt, Swin Transformer, and ViT. As a second exception, the learning rate for ConvNeXt was reduced every 20 epochs by a factor of 0.1.

### MD structure preparation

Three ultra-long MD trajectories of the NiRAN domain present in the RNA-dependent RNA polymerase (RdRp) of SARS-CoV-2 are used to evaluate the transferability of the pretrained models to a structure undergoing significant conformational changes. The trajectories are available from DEShaw research^69^ as DESRES-ANTON-15235444, DESRES-ANTON-15235449, and DESRES-ANTON-15235455, all using a stride of 800 (interval ca. 800 ns). PyMol (http://www.pymol.org/pymol) was used to re-order the atoms in these trajectories. The other 100 short MD trajectories are from the Misato dataset^70^ (PDB codes are listed in supplemental methods, selected trajectories for MD test) with a loading stride of 50 (interval is 4 ns). Each frame is regarded as one PDB structure, and the extraction process followed the same algorithm as in the preparation of the datasets from PDBBind. The extracted test set contains ca. 350,000 entries.

## Results

As mentioned, the residue fragments resemble flexible, macroscopic objects with articulated limbs. Every articulated system (class) has fixed topology, and all 20 amino acids are uniquely identified by their graphs of covalent bonds if all atoms are included. Even if types are obscured, only Ser/Cys are ambiguous in this regard. We therefore did not deem it worthwhile to consider explicit graph neural networks (GNNs) except as positive controls. Here, the in-class heterogeneity differs from that in standard 3D object recognition benchmark sets like ModelNet,^6^ where it has no unique root cause. This is a useful point of reference, and the FEater workflows extend to these data seamlessly. As Table S1 shows, the standard PointNet architecture (here, on the ModelNet40 point clouds sampled from the original surface meshes) offers a good baseline performance broadly in line with literature reports.^11^ In contrast, the spatial similarity across classes is much higher for the protein residues than for ModelNet, and this is where the challenge derives from. Disregarding topology is representative of applications where it is unlikely to help, such as in the discovery of binders to a protein target that rely on new molecular scaffolds or in the design of peptide- or RNA/DNA-based drugs.

### Single-residue-based training

**Figure 5:**
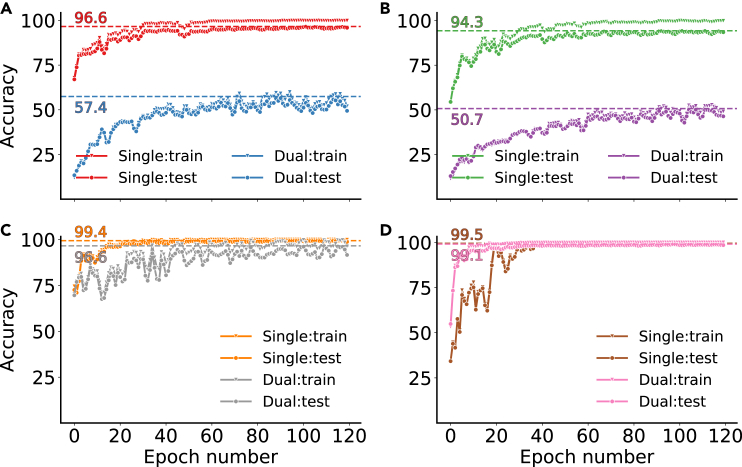
Convergence of different model combinations on balanced one- and two-residue datasets The data shown are classification accuracies for the training data. Training data were balanced by limiting the sample size to 2,000 samples per class. The curves are smoothed by a Savitzky-Golay filter with a window length of 5 and a polynomial degree of 2. Maximum accuracies are indicated by horizontal lines annotated with the respective values. The corresponding plot for the baseline case (blocked data, few samples) is shown in Figure S2. (A) Data for PointNet on coordinates. (B) Data for PointNet on surfaces. (C) Data for VoxNet on voxels. (D) Data for ResNet on Hilbert curves.

**Figure 6:**
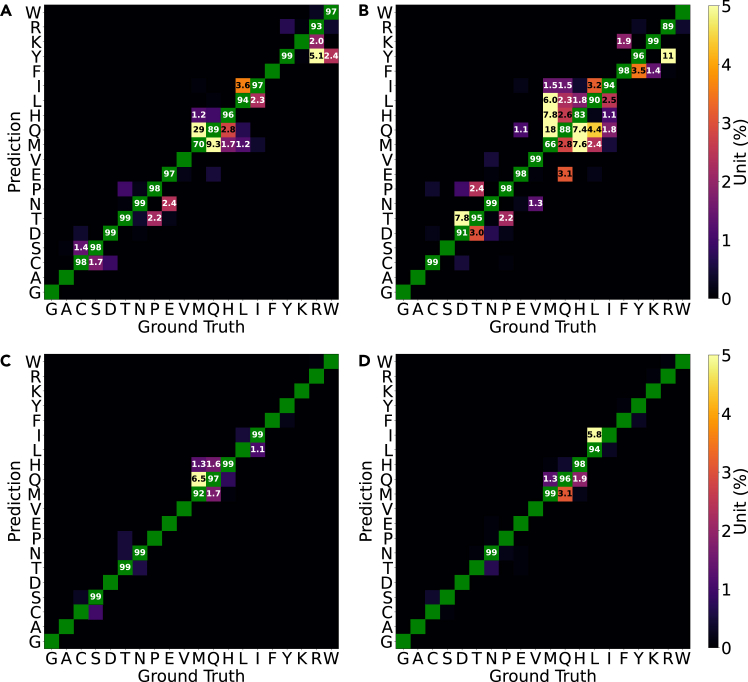
Confusion matrices for the test set when training on FEater-Single Colors are adjusted to enhance the visualization of confusion, with a color scale ranging from 0% to 5%. Confusion rates exceeding 1% are annotated. True positives are highlighted in green, and accuracies below 99.5% are noted. Residues are ordered by the number of atoms (from low to high). (A) Data for PointNet on coordinates. (B) Data for PointNet on surfaces. (C) Data for VoxNet on voxels. (D) Data for ResNet on Hilbert curves.

Individual amino acids differ in their side chains and possess limited flexibility. If the resolution of the spatial information fed to the classifiers is high enough, then it is reasonable to expect that the task is solved comfortably, as seen in Figure 5. However, because we deliberately mask most type-specific information, some confusion between very similar side chains is to be expected. As shown in Figure 6, the number of atoms plays the primary role in these. Several residues, like His/Met/Gln (20), Leu/Ile (22), and Pro/Thr (17), have equivalent numbers of atoms but are not closely related beyond that. Nevertheless, confusion between these is the most common and most severe classification error (most notably Met/Gln). Some amino acids feature flat substructures, such as the phenyl ring in Phe, phenol in Tyr, and guanidine in Arg, which present challenges for PointNet, particularly in molecular surface representations. Interestingly, the confusion between Ser and Cys (which are identical except for one sulfur atom replacing one oxygen atom) is not particularly prominent; this highlights that the models rely mostly on an implicit perception of bond length (here $C_{\beta }$-O-H vs*.* $C_{\beta }$-S-H). This is corroborated by the fact that pairs adopting similar 3D atomic configurations, such as Asp/Asn or Glu/Gln, are seldom prominent and do not feature consistently across models.

As shown in Figure 5, VoxNet demonstrated faster convergence speed and less tendency for overfitting compared to PointNet on either representation, yet ResNet ultimately reached the best performance. As expected, this task is largely trivial for a graph-based representation because these are almost all unique per class and, if constructed only using covalent bonds, independent of conformation. The data for a simple GNN found in Table 1 and Figure S3 make this point (see supplemental methods, choice and training of a graph neural network (GNN) as positive control, for details). The same will hold for most string-based representations. This is in contrast to what we are interested in here, which is to investigate how capable different models are in classifying objects when their conformation (pose) changes. The one-residue data are dealt with well by all models, so we present the models with a more difficult challenge next.

**Table 1:** Performance of general-purpose models trained on the FEater-Single and FEater-Dual datasets

Model/data type	No. of samples	Acc.test	Acc.train
**One-residue cases**			

MPNN + graph	baseline	99.9	100
PointNet + coord.	baseline	67.8	99.8
PointNet + coord.	200	80.1	99.9
PointNet + coord.	400	85.2	99.8
PointNet + coord.	800	92.0	99.7
PointNet + coord.	2,000	96.2	99.9
PointNet + surface	baseline	64.6	96.6
PointNet + surface	200	79.6	100
PointNet + surface	400	86.2	100
PointNet + surface	800	90.0	99.6
PointNet + surface	2000	94.1	99.9
VoxNet + voxel	baseline	86.7	98.8
VoxNet + voxel	200	91.7	99.4
VoxNet + voxel	400	95.1	99.5
VoxNet + voxel	800	98.0	100
VoxNet + voxel	2,000	99.2	100
ResNet + Hilbert	baseline	89.4	100
ResNet + Hilbert	200	94.2	100
ResNet + Hilbert	400	97.8	100
ResNet + Hilbert	800	99.0	100
ResNet + Hilbert	2,000	99.3	100

**Two-residue cases**			

MPNN + graph	baseline	99.4	99.5
PointNet + coord.	baseline	18.9	51.7
PointNet + coord.	200	25.2	48.2
PointNet + coord.	400	34.1	51.5
PointNet + coord.	800	43.3	55.1
PointNet + surface	baseline	13.7	30.8
PointNet + surface	200	25.1	42.0
PointNet + surface	400	30.4	40.0
PointNet + surface	800	35.0	41.4
VoxNet + voxel	baseline	54.0	99.6
VoxNet + voxel	200	63.3	100
VoxNet + voxel	400	81.2	100
VoxNet + voxel	800	92.2	100
ResNet + Hilbert	baseline	77.3	99.9
ResNet + Hilbert	200	84.6	100
ResNet + Hilbert	400	92.2	100
ResNet + Hilbert	800	96.3	100

We trained models with the FEater-Single dataset at 5 training set sizes (baseline, 200, 400, 800, and 2,000 samples per class) to emulate different data scarcities. For FEater-Dual, we used 4 levels (baseline, 200, 400, and 800). Other than having a reduced sample size (117 and 191 for one and two residues, respectively), the baseline data are blocked to reduce overlap between training and test sets (see methods). coord., coordinates.

### Two-residue-based training

**Figure 7:**
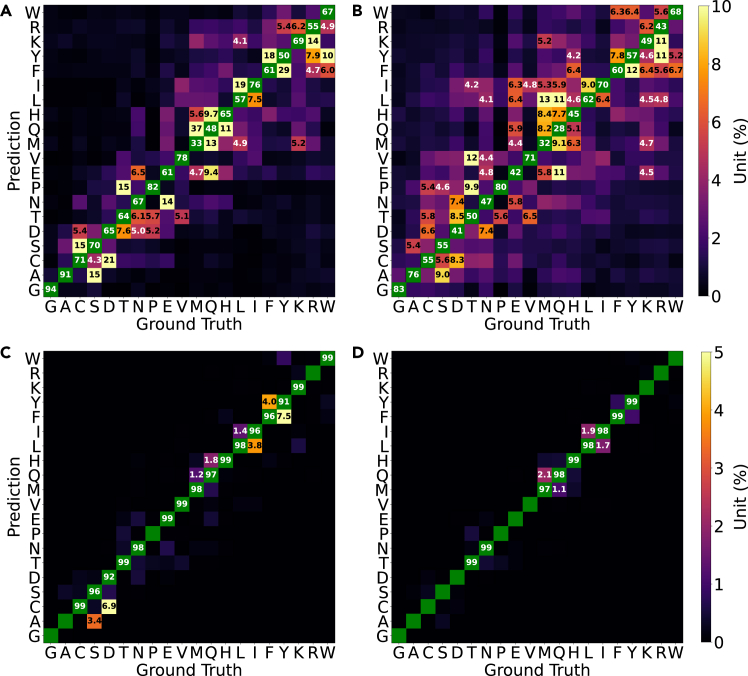
Per-residue confusion matrices for the test set when training on FEater-Dual While there are 400 rather than 20 classes, each class is essentially a 2-tuple, and for ease of presentation, we show here the lumped confusion values per individual position. In all models, the confusion for position 1 is not systematically different from that for position 2 (shown in Figure S4), which justifies the lumping. The color scheme in (C) and (D) is consistent with that in Figure 6. Instead, in (A) and (B), the color scale is adjusted from 0% to 10%, and only confusion rates over 5% are noted explicitly. (A) Data for PointNet on coordinates. (B) Data for PointNet on surfaces. (C) Data for VoxNet on voxels. (D) Data for ResNet on Hilbert curves.

The heterogeneity of shapes for the same label is massively increased if we use stretches of two consecutive residues instead of one. For these two-residue combinations, it arguably becomes paramount for the model to perceive primarily the underlying bond topology from spatially heterogeneous data. As shown in Figure 5, the performance of point-based representations with PointNet drops to around 50%. Similarly, as shown in Figure 7, it is clear that PointNet mislabels many samples and manages to keep low error rates only for side chains that are clearly different in size. There are also prominent negative outliers, especially for pairs involving residues like Met/Gln/Arg, Leu/Ile, and Phe/Tyr, largely following the trends already visible in Figure 6. Evidently, there are architectural limitations that prevent PointNet from abstracting shape from molecular identity.

In contrast, VoxNet and ResNet reached accuracies comparable to the one-residue case. They are expected to perform similarly because Hilbert curves project the same voxel data to simpler patterns, and both solve the task with ease (Figures 7C and 7D). The confusions also follow a similar trend to those of the single-residue problem. This is reasonable because, if we assume that the one-residue task is solvable, then the two-residue task must be solvable as well: because atoms cannot overlap, the 3D patterns of both positions are essentially separable. That said, in practical terms, the training is challenged because the structure of the problem (containing two subtasks) needs to be understood first, for which there is no dedicated mechanism in place. It could be argued that, despite the systematic trends in Figure 5, this is a training and not a model problem. However, Table 1 supports our interpretation, and this is discussed next.

### Emulation of data scarcity

One of the key properties of a learning algorithm is that the outcome improves with more data. As shown in Table 1, all models demonstrate systematic performance improvements with an increase in sample size. Because the number of reasonable conformations for such short fragments is not very large, we created a baseline that not only uses the least amount of samples but also blocks them so that there is reduced overlap, in terms of conformations, between the training and test sets. Even under these conditions, our positive control, an explicit GNN,^71^ solves the problem with ease (see also Figure S3). This is expected because the only non-unique bond graph (Ser/Cys) is distinguished comfortably by the explicit bond distances the model receives as features (see supplemental methods, choice and training of a graph neural network (GNN) as positive control). Furthermore, both the graph and the bond lengths are conformation-independent, so blocking performed in the baseline training has no impact. This is clearly different for the models that receive only spatial information.

In the one-residue case, all models achieved around 100% accuracy on the training set, meaning they possess the complexity to process these geometric features, albeit not necessarily in a transferable manner. The value of the larger training data is only evident from the test set accuracies, which systematically improve with sample size. Differences in test and training set accuracies are a clear indicator of overfitting. For example, the gap between the two shrinks from 20% to 4% for PointNet operating on coordinates with a 10-fold increase in sample size.

Similar to the results in Figure 5, all models showed performance degeneration on the test set when switching from the one- to the two-residue task. Even though the performance of PointNet is poor overall (keeping in mind that the numbers refer to 400 distinct classes), data scarcity affects the training systematically. This gives us confidence that the results in Figure 5 are not too far away from the ceiling for this combination of classifier and input data. Notably, the ResNet + Hilbert curve achieved the highest accuracy and least overfitting in all sample sizes, even under data-sparse conditions. This indicates that the Hilbert curve maps the problem to a more effective representation. We can rationalize this result by the fact that Hilbert curves emphasize locality, which is more closely related to atom connectivity than overall shape.

### Benchmark on community models

We were also curious how modern community models that take 3D or image data as input, but are more specifically designed than PointNet or VoxNet, perform in this task. The list includes a model specifically designed to infer graph-like information from point clouds (DGCNN) and two models associated with molecular science, Gnina and DeepRank-CNN. We emphasize that all the 3D CNNs are architecturally quite similar and differ mostly in orthogonal properties, such as the sizes of their penultimate, fully connected layers.

At the given sample size (1,000 samples per class), all models demonstrated high levels of accuracy in the one-residue dataset. PointNet++, DGCNN, and PAConv all build upon the PointNet architecture by incorporating different hierarchical point feature encoders to construct graphs for topology perception. It is thus expected that they perform much better in the two-residue task than PointNet, and this is borne out by the data. Oddly, PointNet++ performed very well on the raw coordinate representation but less so with the surface representation. We cannot exclude that this particular data point is a training problem, which is indirectly supported by the comparatively low training accuracy even for FEater-Single (worst of all model combinations in Table 2). PAConv maintained excellent performance across both types of representations.

**Table 2:** Performance of different models trained with 1,000 samples per label

Model/data type	No. of parameters (M)	Acc. test	Acc. train
**One-residue cases**			

PointNet + coord.	1.6	94.0	100
PointNet++ + coord.	1.5	98.3	100
DGCNN + coord.	1.8	97.2	100
PAConv + coord.	2.4	99.6	100
PointNet + surface	1.6	91.2	99.8
PointNet++ + surface	1.5	86.1	88.5
DGCNN + surface	1.8	99.3	99.9
PAConv + surface	2.4	95.4	99.7
VoxNet + voxel	8.9	98.6	100
DeepRank-CNN + voxel	0.15	93.4	94.5
Gnina + voxel	0.4	96.4	97.3
ResNet + Hilbert	11.2	99.3	100
ConvNeXt + Hilbert	27.8	94.1	95.7
ConvNeXt Iso + Hilbert	21.7	97.5	99.9
Swin Tran. + Hilbert	27.5	96.8	100
ViT + Hilbert	85.3	95.2	99.7

**Two-residue cases**			

PointNet + coord.	1.7	34.0	39.7
PointNet++ + coord.	1.6	99.4	99.4
DGCNN + coord.	1.9	89.0	90.1
PAConv + coord.	2.5	99.4	99.4
PointNet + surface	1.7	41.4	47.3
PointNet++ + surface	1.6	55.6	58.0
DGCNN + surface	1.9	99.4	99.7
PAConv + surface	2.5	99.4	99.8
VoxNet + voxel	9.4	82.2	94.4
DeepRank-CNN + voxel	0.18	47.7	50.2
Gnina + voxel	3.6	82.2	92.4
ResNet + Hilbert	11.4	97.1	100
ConvNeXt + Hilbert	28.1	94.9	100
ConvNeXt Iso + Hilbert	21.9	94.7	100
Swin Tran. + Hilbert	27.8	98.1	100
ViT + Hilbert	85.6	90.3	100

Acc., accuracy; coord., coordinates; Tran., transformer.

Two 3D CNNs, Gnina and VoxNet, demonstrated the largest amount of overfitting in the two-residue dataset at 1,000 samples per label. This is noteworthy because it suggests that 3D CNNs are inherently more prone to learn non-transferable ways of solving the training problem. The remaining 3D CNN, DeepRank-CNN, is, in our instantiation, the smallest model, with only 84 neurons in the penultimate layer, which is likely what causes it to suffer for the 400 classes of FEater-Dual. The image-based models achieved higher training set accuracies and demonstrated lower performance loss to this dataset switch. Interestingly, recent models like ConvNeXt, Swin transformer, and ViT did not outperform the baseline ResNet architecture, which is consistent with the hypothesis that this performance gain is primarily a property of Hilbert curves. We emphasize that all models are evaluated with fixed architecture sizes, which can be another important performance differentiator, as highlighted by the comparison of Gnina with DeepRank-CNN.

### Benchmark on data retrieval

**Figure 8:**
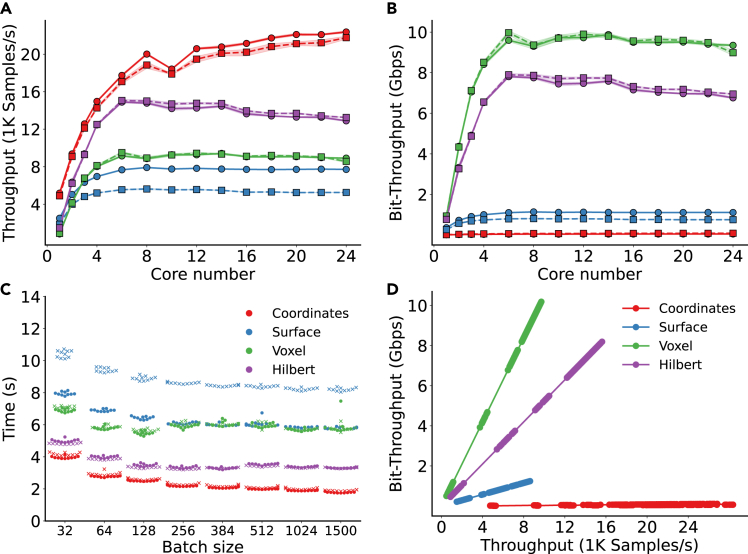
Benchmark of iterating data on subsets taken from the FEater-Single and FEater-Dual datasets Each subset contains 50,000 samples. (A) Sample throughput for the four molecular representations as the function of the core number employed in the data loaders. FEater-Single is marked by circles and FEater-Dual by squares (same color legend as in D). (B) Same as (A) but plotting the bit throughput. (C) Iteration time as a function of batch size for different data types. (D) Bit throughput as a function of the sample throughput for different data types. The configuration of the benchmark system was Intel Core i9 13900K@24 cores, hyperthreading off, DDR5 5200 MHz 32GB × 2, SSD - WD SN850X 4TB, OS - Ubuntu 22.04, Chipset - ASUS Z790.

Since Hilbert curves and 3D voxels are homogeneous and data intensive (each sample contains ∼16,000 and ∼32,000 floating point numbers, respectively), the required throughput of bits is significantly higher than for the heterogeneous, coordinate-based datasets. Data retrieval can be limiting, depending on the hardware. Figure 8 assesses the parallel performance of this step. The strong scaling results in Figures 8A and 8B show that parallel disk access saturates quickly and is often well below the ceiling for bit throughput (i.e., at least as high as for the voxel data in Figure 8B). Coordinate-based representations follow a *retrieval* – *padding/sub-sampling* – *shuffling* routine during data extraction. This creates on-the-fly overhead, which, for a 1,500 point set, becomes the bottleneck of data extraction. While this matters little for raw atomic coordinates (due to the extremely low bit size, almost flat curve in Figure 8D), it is the primary reason why surface-based representations fare poorly in both throughput tests and why they are consistently the slowest (Figure 8C).

### Transferability of models to MD trajectories

The samples in the FEater datasets are extracted from static structures determined experimentally. This means that their notion of dynamic changes is only indirect. We thus wanted to inquire what happens if the samples considered are subject to explicit, time-dependent evolution.

**Figure 9:**
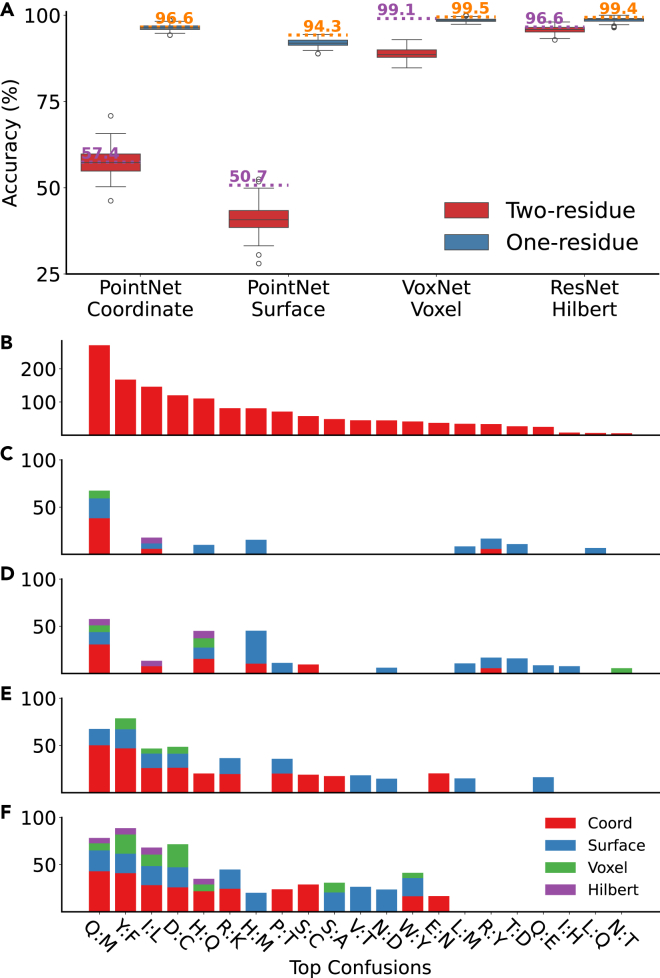
Performance on MD data and top confusion across different trainings (A) Accuracies of the pretrained models on MD datasets shown as Tukey-rule boxplots across 103 trajectories. The short dashed horizontal lines and text provide the respective models’ accuracy on the original FEater test sets. (B) Top confusion aggregated from both the original test sets and trajectory-based test sets. (C–F) Bar plot of the most frequent, per-residue confusion in different aggregated samples: testing on FEater one- (C) and two-residue datasets (E) or the same for MD data (D and F). The confusion pairs are ordered by the overall population, measured as the numerical sum of individual percentages. The color legend in (F) applies to all.

Figure 9A demonstrates that the models can assign the correct label to different conformations of fragments from MD data with accuracy comparable to the FEater test sets. This means that pretrained models, exemplified here for the same combinations as in Figure 5, do translate to MD data. Only two trainings, on two-residue-based surface and voxel representations, showed ca. 10% performance degeneration. In contrast to the static PDB structures, from where these fragment datasets were built, during MD simulations, conformational fluctuations are observed continuously in time, in particular for surface residues. The consistency of the results with those from the FEater datasets supports the idea that augmenting data-driven drug discovery workflows with MD structure ensembles might help in abstracting irrelevant aspects in the heterogeneity of static measured structures.

## Discussion

We systematically evaluated the robustness of different molecular representations in a recognition task to differences in the 3D shapes of the recognized objects. By framing the flexibility of molecular fragments as a 3D object classification task, we hope to provide a benchmark for featurization and models that is challenging enough but unequivocally labeled, easily balanced, and straightforward to work with. FEater establishes a standard workflow to compare the combination of different models and different input data and offers a platform for rigorous performance comparison. FEater contains two large-scale datasets to systematically train and evaluate models and provides a user-friendly and efficient interface that stores 3D features and allows access to entries in constant time.

The FEater benchmark sets differ from standard computer vision benchmark sets in that every class is made heterogeneous purely by conformational changes of a fragment with fixed molecular topology. Most of these topologies are unique, albeit with high mutual similarity, which is why GNNs are able to address the task with ease. In computer vision applications, this most closely resembles scenarios like recognizing different entities (e.g., humans, animals, robots) in 3D scenes when they are captured in different poses. This refers to both individuals within the same species (in analogy, Cys/Ser have the same topology but different “limb” lengths, while others are very similar, like Phe/Tyr) and across species. In contrast, most diversity in ModelNet comes from different basic shapes mapping to the same class. Thus, FEater incentivizes shape abstraction by topology inference, whereas ModelNet might favor the learning of discrete subclasses mapping to the same label. It should thus be interesting to see how models perform in both tasks or to conjoin the benchmarks sets for training.

A second issue to highlight is the lack of ambiguity in the FEater labels. In datasets like MNIST or ModelNet, there will be few cases with questionable labels (e.g., a vase can be a flower pot). However, in molecular science, the task is often to predict quantities that are theoretically measurable, based on training data that can be predictions themselves.^72^^,^^73^ But even if experimental data are available, errors of many kind can easily creep into the labels: statistical measurement errors, systematic errors (such as when a molecule is in equilibrium in solution but the label is assumed to hold indiscriminately for all forms), etc. The more complex the measurement, the less clear it becomes that it is strongly associated with a given sample. This effect is already quite pronounced in *in vitro* data of target binding affinities, and the often-observed lack of generalizable insights from ML models might be influenced by this.^74^ FEater allows separating the data scarcity component from concerns about the labels.

At the single-residue level, we found that all four distinct featurizations allowed general-purpose models to be trained successfully. It is important that these models were not designed for biomolecular studies. Similarly, some more recent community models also solved the two-residue problem with ease despite their lack of specializations inspired by molecular science. We thus demonstrate that mere 3D geometries do encode the chemical identity of molecules in a way that is perceivable by general-purpose ML models. By establishing the transferability of pretrained models to datasets that encode real dynamics rather than just heterogeneity, we corroborate the successful recognition of molecular fragments as flexible objects with well-defined motion profiles. Clearly, our benchmarks offer some simplifications compared to many real-world applications, such as a completely homogenized scale and automatic centering. Just as with the unambiguous labels discussed above, this can be an advantage, as it separates different stages of perception and understanding and can thus more easily probe where generalizability is lost.

Generalizability, not training set accuracy, is the biggest problem in data-sparse regimes. It is thus interesting to ask what we can learn in terms of how to tackle this issue from the comparisons presented here. First, the parameter richness is a universal problem, and modern ML models can no longer be analyzed in terms of parsimony: they are all needlessly complex. Due to their specific designs, not all parameters are useful, meaning that models with >1M parameters can struggle with this relatively simple perception task, while others of comparable size excel. It would, in future work, be of interest to perform a reductionist pruning of a selected architecture to test whether generalizability can be systematically recovered by such pruning. Complementarity of predictions (ensemble learning) is a common way to increase robustness. We tested whether a similar logic can be used by joining models to benefit from complementary representations, but the results were underwhelming (Table S2; Figure S5). We did not explore feature or label noising^75^ but consider it to be a promising avenue. One of the issues it presents is the introduction of new hyperparameters.

There are three additional points to raise. First, not all of the models can be trained effectively with out-of-the-box parameters (see methods, model training). In real applications this can be difficult to diagnose, and attempting to establish systematic trends with data sparsity as in Table 1 is one of few ways to do so. Second, while the architectures of models are complex, mostly in a parameter sense, the ones we chose here are far from offering general artificial intelligence. If the research is hypothesis driven, then this is not a downside, of course, but it does mean that details of the featurization continue to matter. The difference in results between PointNet and PointNet++^11^ in Table 2 is a good illustration of this remark: the latter is specifically designed to respect spatial localities, which here translate to covalent relations between atoms. Third, maintaining performance from the one- to the two-residue case might report on how well a model performs implicit task segmentation in the chosen feature space. This comprehension of modularity is an important property for working with crowded 3D scenes.

Drug discovery or protein engineering are data-sparse fields: efforts have been undertaken and are underway to improve this somewhat,^76^ but it will remain true that, for example, only ∼1,000s of small molecules are approved drugs. Even relatively simple properties like solubility or pKa values are not widely available as experimental measurements and are thus hard to train models on. Consequently, much effort has been invested toward replacing expensive, theoretical predictions with ML ones,^73^ a trend also observable in weather forecasts.^77^ Complex ML models frequently pick up trivial aspects of the input features that have low predictive value, as observed in many ML scoring functions used in computational drug discovery.^78^^,^^79^ By uncoupling spatial information from explicit topological and type information, the FEater benchmark sets prevent many of these trivial aspects from influencing the learning. Our results and the datasets offer guidance on what it can mean in practice to train models of this scope with only tens of samples per class, which is a common scenario in drug discovery applications. In applications where spatial information is paramount, such as interface or binder prediction, FEater will allow the establishment of a convenient baseline for the model of choice. We can envision the construction of similar datasets containing “spatially continuous” fragments where not all atoms belong to a single, continuous piece of a molecule to more specifically aid such applications.

## Resource availability

### Lead contact

Further information and requests for resources should be directed to the lead contact, Andreas Vitalis (a.vitalis@bioc.uzh.ch).

### Materials availability

This study did not generate new unique reagents.

### Data and code availability

The FEater datasets are publicly available at Zenodo.^80^ All source code in this research is hosted on GitHub (https://github.com/miemiemmmm/FEater) and has been archived at Zenodo.^81^ All models are implemented and trained in PyTorch 2.1.1^82^ under Python 3.9.18. PyTraj^83^ is used to read the protein topology and trajectory.

## Acknowledgments

We are grateful to Amedeo Caflisch for helpful discussions and continued support. This work was supported in part by grant 189363 from the 10.13039/501100001711Swiss National Science Foundation to Amedeo Caflisch.

## Author contributions

Conceptualization, investigation, writing – review & editing, A.V. and Y.Z.; methodology, software, formal analysis, visualization, writing – original draft, Y.Z.; resources, supervision, A.V.

## Declaration of interests

The authors declare no competing interests.

## Supplemental information

Document S1. Figures S1–S5, Tables S1 and S2, Algorithms S1–S4, and supplemental methods

Document S2. Article plus supplemental information
