# Supplementary material for: Benchmarking the robustness of the correct identification of flexible 3D objects using common machine learning models
Source: Patterns (N Y). 2025 Jan 10;6(1):101147. doi: 10.1016/j.patter.2024.101147 (PMC11783895; doi:10.1016/j.patter.2024.101147)
Supplement: Document S1. Figures S1–S5, Tables S1 and S2, Algorithms S1–S4, and supplemental methods [file mmc1.pdf]

**Patterns, Volume *6***

## Supplemental information

# Benchmarking the robustness of the correct identification of flexible 3D objects using common machine learning models

**Yang Zhang and Andreas Vitalis**

# Supplemental Information

## Supplemental Methods

### Voxel generation

The details on the mathematical framework to estimate the volumetric density contribution for a given atom are as follows (also see "Methods, Voxel generation" in the main text):

$$T_{ijk,n} = \frac{1}{\sigma\sqrt{2\pi}} exp(-\frac{d^2_{ijk,n}}{2\sigma^2}) \quad (1)$$

where $T_{ijk,n}$ is the value of the voxel at position $(i, j, k)$ of the temporary Gaussian map $T_n$, $d_{ijk,n}$ is the distance between the voxel and atom $n$, and $\sigma$ is the smoothness factor of the property distribution. The final Gaussian map $V_n$ for atom $n$ is generated by the following equation:

$$V_{ijk,n} = \frac{T_{ijk,n}}{\sum_{i,j,k} T_{ijk,n}} \times w_n \quad (2)$$

where $w_n$ is the weight of the $n$-th atom, and $\sum_{i,j,k} T_{ijk,n}$ is the sum across the temporary Gaussian map, $T_n$. The total volumetric density is the superposition of the contributions from all atoms.

### Selected trajectories for MD test

For the data in Figure 9 in the main text, the following list of 100 trajectories in the Misato MD dataset (identified by their corresponding PDB codes) was employed for the inference on pretrained models.

```
6qae, 6p14, 6ink, 9icd, 6n3y, 6ew3, 6j3p, 6eis, 6sfj, 6eq2,
6rqk, 6eab, 6n78, 6gn1, 6o9d, 6rml, 6eru, 6gi6, 6r1d, 6sze,
6gip, 6miv, 6ob0, 6moo, 6gjl, 6qtx, 6ql1, 6h7z, 6dq4, 6g1w,
6gw1, 6pyd, 6isd, 6ugq, 6ftp, 6m8y, 6htp, 6o94, 6mt4, 6oir,
6fac, 6qsz, 6f26, 6hzv, 6f6r, 6hzp, 6ewe, 6ekn, 6ow7, 6fvn,
6fiv, 6oe1, 6fnq, 6ma3, 6dy7, 6j10, 6g2e, 6gg8, 6ffg, 6f2n,
6g8j, 6el5, 6m8e, 6qi7, 6oa3, 6i8z, 6f8g, 6ugo, 6dz3, 6e5s,
6g2n, 6mlh, 6e06, 6jao, 6ee6, 6gfz, 6fs1, 6t6a, 6frf, 6g6y,
7abp, 6f3f, 6gr7, 6qmj, 6nk0, 6np2, 6gpb, 6h7y, 6e4w, 6qts,
6o5g, 6gxu, 6f86, 6ezq, 6drt, 6ey8, 6ht1, 6pi1, 6hvw, 6j9w
```

### Choice and training of a graph neural network (GNN) as positive control

We wished to supplement the data in Table 1 in the main text with a positive control. As is explained at the beginning of Results, the graphs formed by the covalent bonds of the 20 standard amino acids are all unique, with the exception of Ser and Cys. This holds if hydrogen atoms are considered explicitly. Thus, a GNN that receives the (correct) bond graph has, for the most part, the simple task of mapping discrete input classes to discrete output classes. In practice, a graph might be estimated from coordinates using a distance threshold, and we did so to emulate the more general case (with a threshold of 2.0Å). This is sufficient for capturing all true covalent bonds but might introduce sporadic artificial bonds between two hydrogen atoms. Aside from this

variability, the graph is annotated with features, here atomic coordinates as node attributes and inter-atomic distance as edge attributes.

These featurizations of the FEater-Single and FEater-Dual datasets were precalculated and fed to a Message Passing Neural Network. The specific model can be found in `https://github.com/miemiemmmm/FEater/tree/main/feater/models`. The training was performed under the Deep Graph Library (DGL) framework. It proceeded straightforwardly (see Figure S3 below) and reached test set accuracies beyond 99.5% very quickly. As a sanity check, we also trained a model with a poor threshold of 1.5Å. This is very close to the value around which single C-C bonds fluctuate. Thus, the graphs will have sporadic variability in them, and the task for the GNN is much harder: it will have to use the annotated vertex (atom) features (coordinates) to improve its prediction accuracy (see caption to Figure S3).

## Access to standard computer vision benchmarks

The framework is easily extensible to benchmark sets used in computer vision tasks. ModelNet40 is such a standard benchmark set containing 40 classes of everyday objects. To use these data, which are also stored in HDF5 format in surface representation, the workflows provided by the FEater repository require only a single modification: the inclusion of a scaling operation, which we provide. The results of training different point cloud-based models are summarized in Table S1.

## Algorithms

**Algorithm S1** Construction of FEater-single dataset

```
Input: Protein list C
Constant: 20 label types L
for c in C do
  p = load_topology(c)
  N = get_residue_number(p)
  for i = 0 to N do
    Get residue R at position i: R = get_residue(p, i)
    Prepare CAMPARI input files
    if R in L then
      Write R to a temporary PDB file
      Fix the PDB of R by CAMPARI
      Save CAMPARI outputs
    end if
  end for
end for
```

**Algorithm S2** Construction of FEater-dual dataset

```
Input: Protein list C
Constant: 400 label types L
for c in C do
  p = load_topology(c)
  N = get_residue_number(p)
  for i = 0 to N − 1 do
    Get residue at position i: r = get_residue(p, i)
    Get residue at position i + 1: r′ = get_residue(p, i + 1)
    if r not connected to r′ then
      continue
    end if
    Get two-residue R at i: R = get_two_residue(p, i, i + 1)
    Prepare CAMPARI input files
    if R in L then
      Write R to a temporary PDB file
      Fix the PDB of R by CAMPARI
      Save CAMPARI outputs
    end if
  end for
end for
```

**Algorithm S3** Voxel generation

**Input:** Atom coordinate set $A[N, 3]$, weights $w[N]$, number of atoms $N$, grid dimension $d[3]$, grid spacing $s$, sigma $\sigma$
$n = d_1 \times d_2 \times d_3$
**Initialize:** Result 3D voxel in an 1D array $V[n]$
**Align $A$ with voxel:** $A$ -= $d/2$ - center_of_geometry($A$)
**for** $i = 0$ **to** $N$ **do**
  **Initialize:** Temporary 3D voxel $V'[n]$
  **CUDA parallelization of for loop**
  **for** $j = 0$ **to** $n$ **do**
    **Initialize:** Temporary coordinate $c'[3]$
    $c'$ = voxel_coordinate($j$, $d$, $s$)
    $d'$ = distance($A_i$, $c'$)
    $V'_j$ = gaussian($d'$, $\sigma$, $w_i$)
  **end for**
  **Synchronize CUDA kernel**
  **Normalize $V'$:** $V'$ = ($V' \times w_i$) / sum($V'$)
  **CUDA parallelization of for loop**
  **for** $j = 0$ **to** $n$ **do**
    $V_j$ += $V'_j$
  **end for**
  **Synchronize CUDA kernel**
**end for**
**Reshape $V$ to 3D:** $V$ = reshape($V$, $d$)
**Output:** $V$

**Algorithm S4** Mapping 3D voxel to 2D Hilbert curve

**Input:** 3D Voxel $V[32, 32, 32]$
**Initialize:** Result 2D image $img[128, 128]$
**Initialize:** 3D Hilbert curve $H^{3D}[32 * 32 * 32, 3]$, 2D Hilbert curve $H^{2D}[128 * 128, 2]$
**Initialize:** Temporary 1D Hilbert curve $H^{1d}[32 * 32 * 32]$
**for** $i$, $p$ in enumerate($H^{3D}$) **do**
  $v_i$ = $V_p$
**end for**
$s$ = array_split($v$, 128*128)
$s'$ = max_pooling($s$)
**for** $i$, $p$ in enumerate($H^{2D}$) **do**
  $img_p$ = $s'_i$
**end for**
**Output:** $img$

## Supplemental Tables

Table S1: **Example training results on ModelNet40 data**. As explained in the main text and in "Supplemental Methods, Access to standard computer vision benchmarks" above, the FEater repository allows seamless access to existing benchmark sets, such as ModelNet. The table shows training and test set performances for standard models on ModelNet40, using the recommended splits. The models are straightforwardly trainable to very high fidelity. The test set accuracies are slightly below what is found in the literature for these models, but this is not surprising given that we used only a single, fixed-length training (120 epochs) without dataset-specific hyperparameter optimization. The most common confusions are intuitive and shared by all models: for example, "vase" *vs* "flower pot", "night stand" *vs* "dresser", and "table" *vs* "desk" are in the top 5 most common confusions (by pair) for all three models listed below.

| MODEL | ACC. TEST | ACC. TRAIN |
|---|---|---|
| POINTNET | 84.6 | 98.4 |
| DGCNN | 86.3 | 99.5 |
| PACONV | 85.5 | 99.4 |

Table S2: **The accuracy of fused models**. By connecting the penultimate (and fully connected) layers of two different models to the same output layer, we created a series of fused models (see main text). All fused models conjoin two different base representations with each other, and we used Gnina for voxels, PointNet for point clouds on both coordinates and surface vertices (referred to as "PointNet*" below), and ResNet for 2D Hilbert curves. All training was done under "Baseline" conditions, with sparse data and blocked training and test sets. This setting offers the maximum amplitude for detecting a synergistic effect. A visual representation of the same data is shown as part of Figure S5.

| MODEL1 | MODEL2 | ACCURACY |
|---|---|---|
| GNINA | POINTNET | 79.0(100) |
| GNINA | POINTNET* | 80.8(100) |
| GNINA | RESNET | 68.5(100) |
| POINTNET | POINTNET* | 68.2(100) |
| POINTNET | RESNET | 68.8(100) |
| POINTNET* | RESNET | 71.6(100) |
| GNINA | POINTNET | 33.9(100) |
| GNINA | POINTNET* | 25.1(95.3) |
| GNINA | RESNET | 61.3(100) |
| POINTNET | POINTNET* | 24.9(46.9) |
| POINTNET | RESNET | 64.4(100) |
| POINTNET* | RESNET | 63.3(100) |

## Supplemental Figures

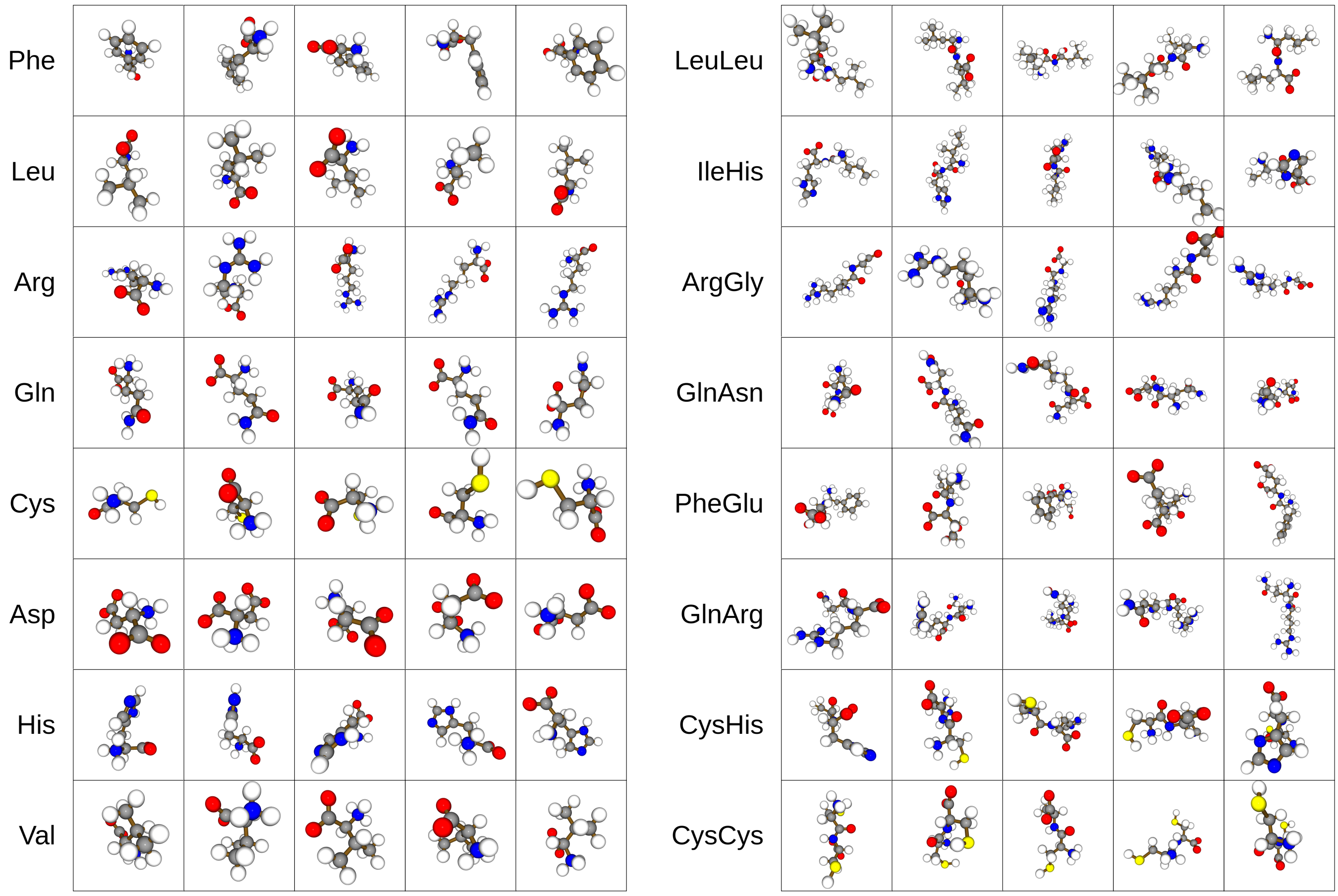


Figure S1: **Auxiliary 3D visualizations of the two datasets**. Several example structures in the FEater-Single and FEater-Dual datasets are shown as molecular stick and ball representations (same as Figure 2 in the main text).

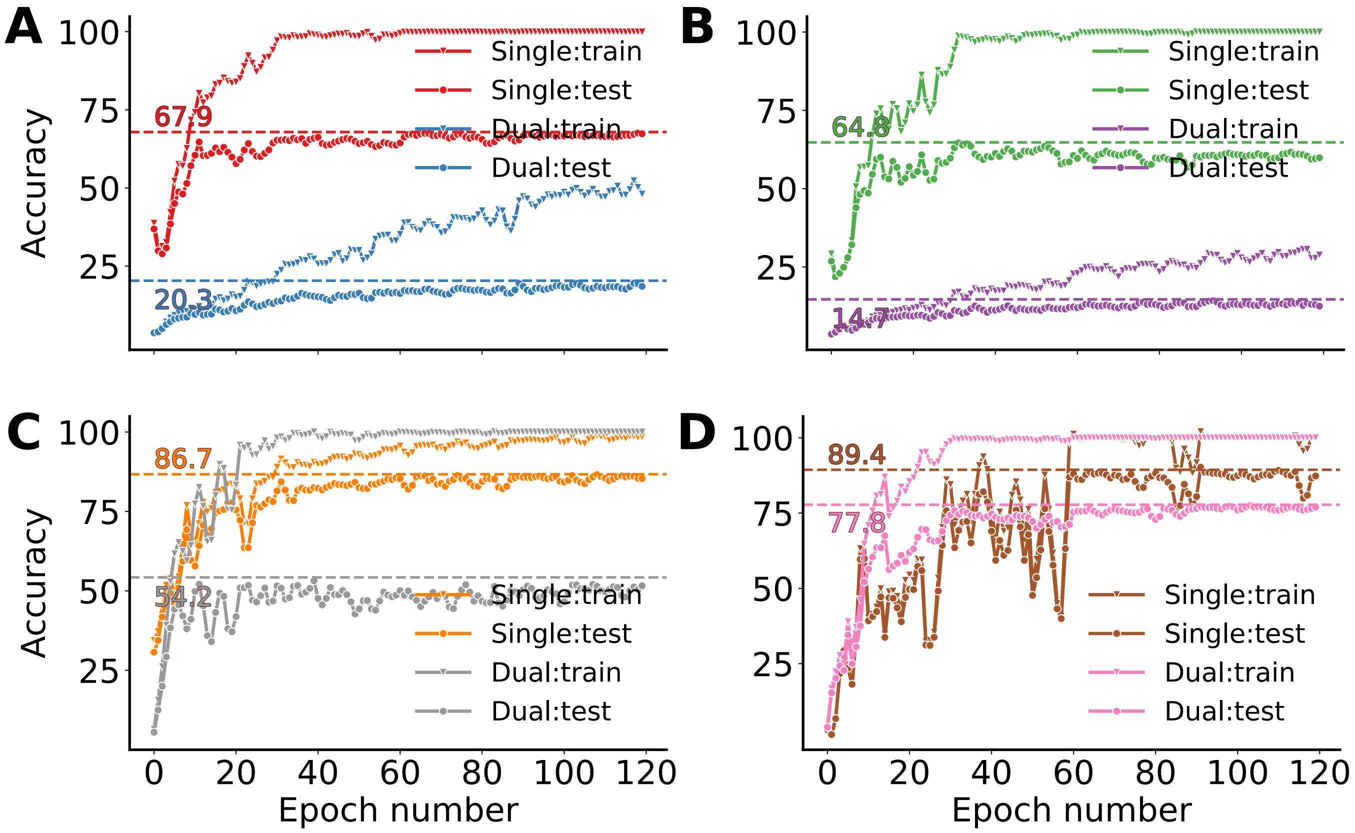


Figure S2: **Model training under stringent conditions**. To provide a baseline expectation for performance in a regime where data are both sparse and offer limited coverage, we followed the procedure described in the main text, see "Methods, Construction of blocked dataset." This leads to the training relying on only 117 (FEater-Single) and 191 (FEater-Dual) samples per class with a significantly reduced conformational overlap between test and training sets. The figure is analogous to Figure 5 in the main text, with the exception that all data are for these stringent "Baseline" settings referred to in Table 1 in the main text. Arguably, the training set accuracies in panels A and B for FEater-Dual might continue to improve with more training, but the test set performances have already encountered a clear plateau in both cases. **A**. Data for PointNet on coordinates. **B**. Data for PointNet on surfaces. **C**. Data for VoxNet on voxels. **D**. Data for ResNet on Hilbert curves.

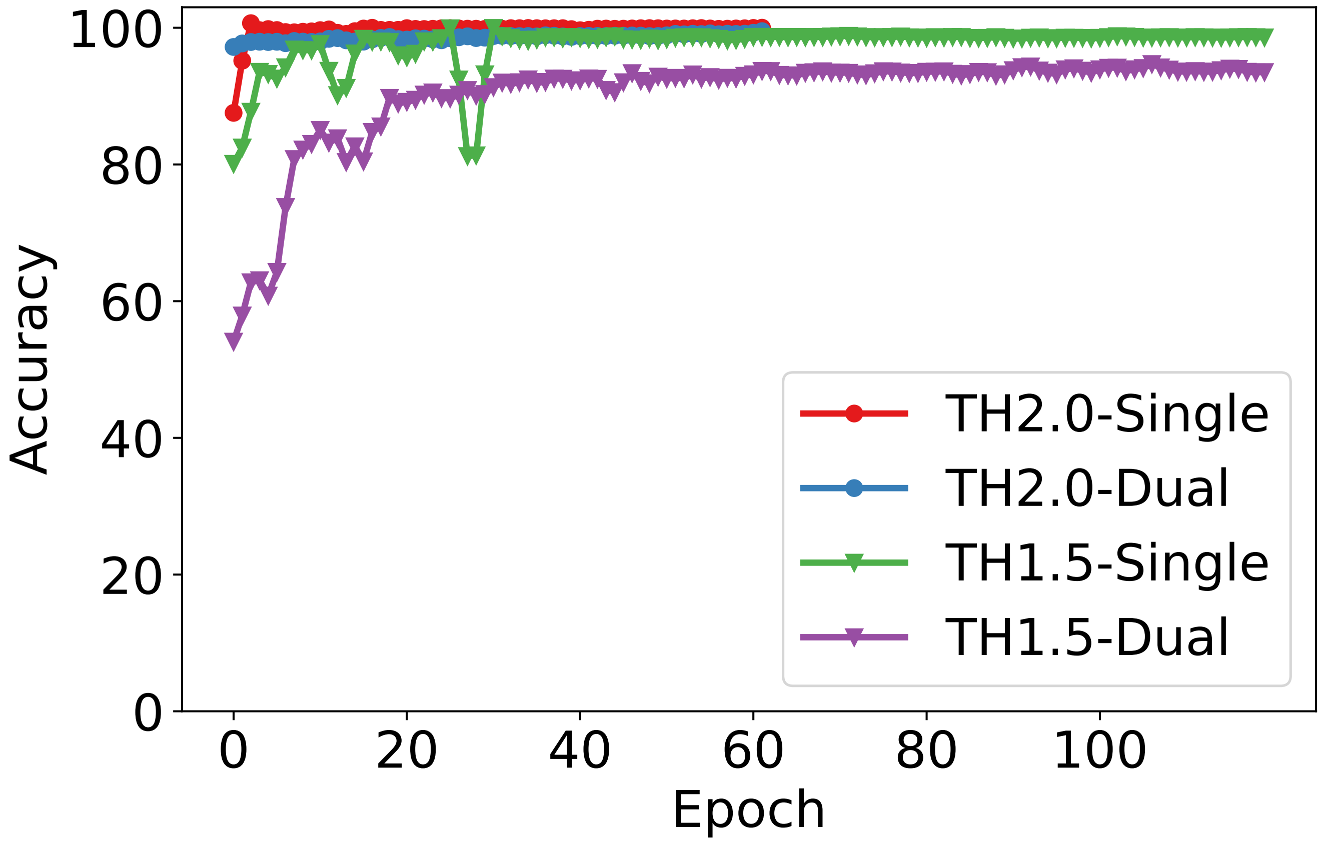


Figure S3: **Training of a message-passing network as a positive control**. We plot test set accuracy as a function of the number of epochs. See "Supplemental Methods, Choice and training of a graph neural network (GNN) as positive control," for details. "TH2.0" refers to using a threshold of 2.0Å to heuristically determine the bond graph, while "TH1.5" refers to using a threshold of 1.5Å instead. With the 2.0Å threshold, the models solve the problem with ease, and we extended training only until the $60^{th}$ epoch. The smaller threshold is a deliberate control to ensure that no other information than spatial coordinates is responsible for this high training efficiency and excellent performance. In this case, single C-C bonds will "flicker", leading to largely random graph heterogeneity, which makes the problem substantially more complicated. It is consistent that the top confusion for the "TH1.5" was between Ile and Leu, two residues with aliphatic side chains and the same number of carbon atoms.

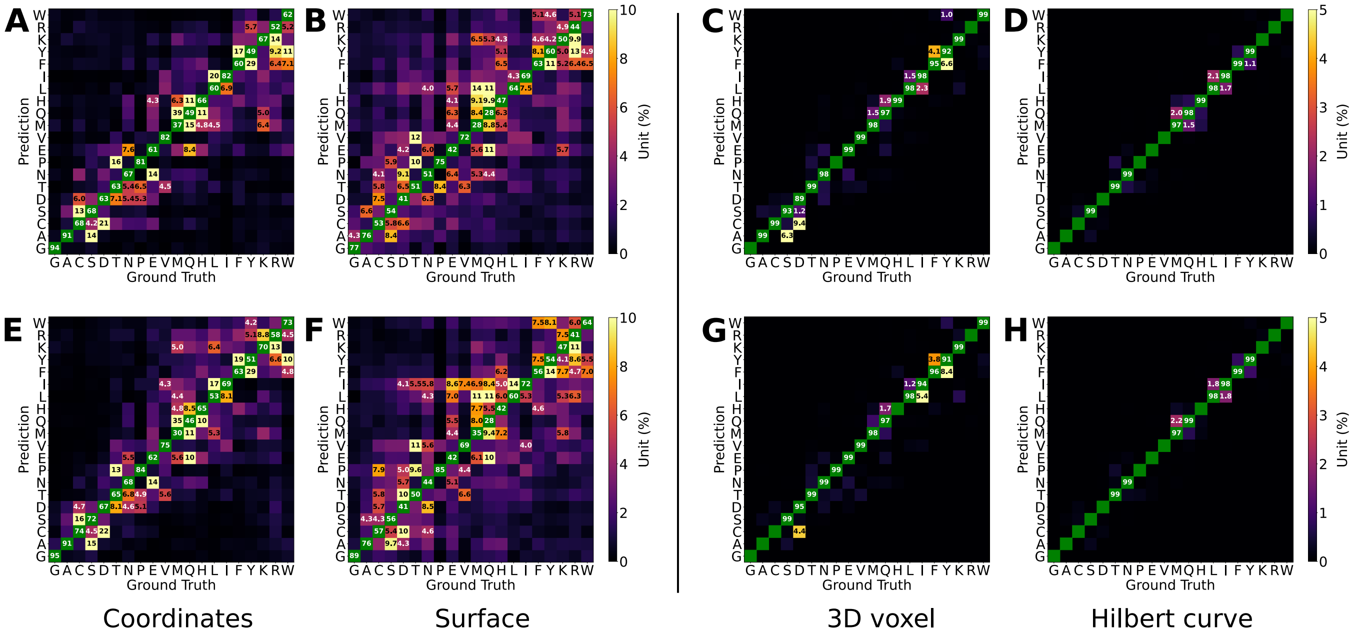


Figure S4: **Differences in confusion between different positions in the two-residue data set**. The individual panels are analogous to Figure 7 in the main text but show the raw data per position. Figures A-D represent position 1 and E-H position 2, while the columns distinguish the model/feature combinations (left to right, same as A-D in Figure 7 in the main text).

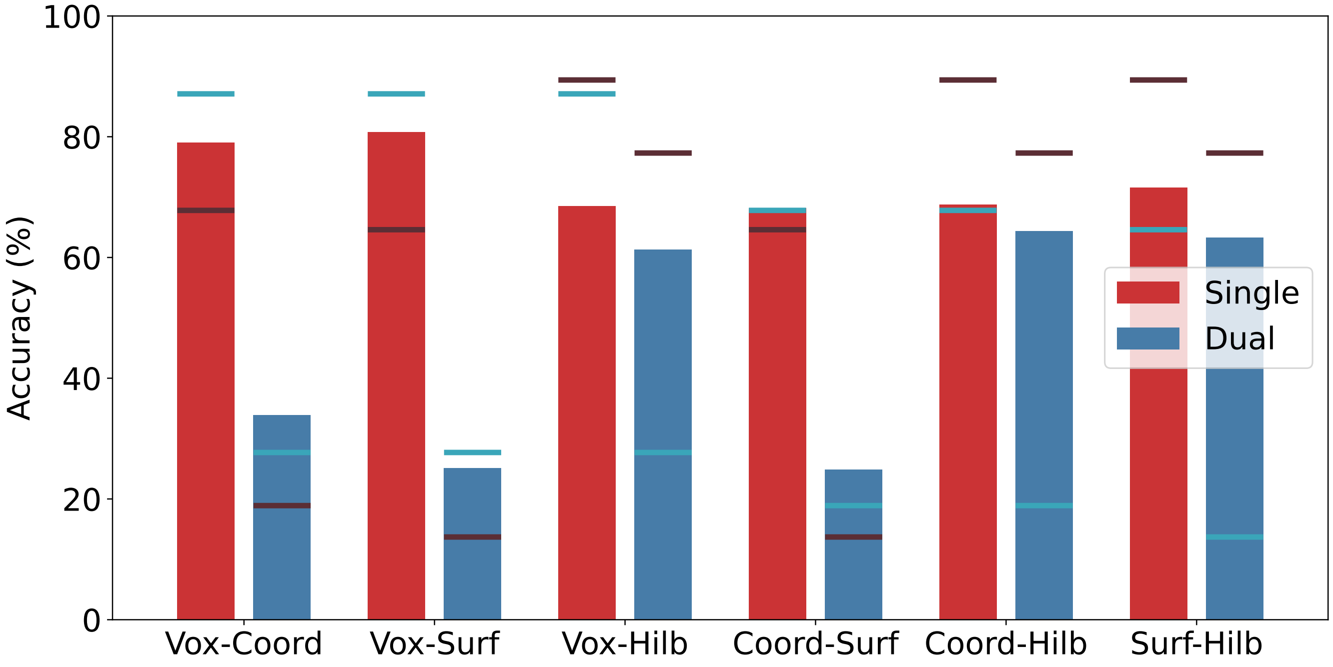


Figure S5: **Performance of fused models compared with their parent models**. The bar graphs show the performance on both data sets (same as Table S2) while the horizontal lines give the test set accuracies for the parent models: cyan for the first, dark red for the second. The data for the parent models are the same as those reported in Table 1 in the main text. “Surf” and “Coord” corresponds to PointNet, “Vox” to Gnina, and “Hilb” to ResNet. In the majority of cases, the performance of the fused model is below that of the superior parent, indicating a lack of synergy. We observe a modest synergistic effect only in cases where both parent models are poor (like “Vox-Coord” on FEater-Dual). Overall, all fused models appear to have a low ceiling near 80% which is surprising, particularly for FEater-Single.
